# Effects of Fomepizole on Acetaminophen Oxidative Metabolism: A Randomized, Crossover Study in Human Volunteers

**DOI:** 10.1002/cpt.70379

**Published:** 2026-07-14

**Authors:** Sidharth Anand, Nicholas A. Buckley, Paul Stathakis, Laura James, Larry Parker, Mitchell R. McGill, Angela L Chiew

**Affiliations:** ^1^ Faculty of Medicine The University of New South Wales Sydney New South Wales Australia; ^2^ Faculty of Medicine and Health The University of Sydney Camperdown New South Wales Australia; ^3^ NSW Health Pathology, Prince of Wales Hospital Randwick New South Wales Australia; ^4^ Department of Pediatrics, College of Medicine University of Arkansas for Medical Sciences Little Rock Arkansas USA; ^5^ Acetaminophen Toxicity Diagnostics, LLC Little Rock Arkansas USA; ^6^ Department of Environmental Health Sciences, Fay W. Boozman College of Public Health University of Arkansas for Medical Sciences Little Rock Arkansas USA; ^7^ Department of Pharmacology and Toxicology, College of Medicine University of Arkansas for Medical Sciences Little Rock Arkansas USA; ^8^ Department of Clinical Toxicology Prince of Wales Hospital Randwick New South Wales Australia

## Abstract

Acetaminophen overdose can cause liver injury. Hepatotoxicity results from *N*‐acetyl‐*p*‐benzoquinone imine (NAPQI), generated via cytochrome P450‐2E1 (CYP2E1). Acetylcysteine is the mainstay of treatment, but efficacy may be reduced with delayed administration or large ingestions. Fomepizole, a CYP2E1 inhibitor, reduces NAPQI when co‐administered with acetaminophen, but its clinical relevance remains uncertain. The objective was to evaluate the effect of fomepizole administered 2 hours after acetaminophen ingestion in immediate‐ and modified‐release overdose. In a randomized crossover study simulating overdose, healthy volunteers received ~≈80 mg/kg of either immediate‐ or modified‐release acetaminophen followed by intravenous fomepizole or placebo 2 hours later. Volunteers crossed over after 2 weeks. Urine and serum were collected over 24 hours to measure acetaminophen, non‐toxic metabolites (APAP‐Glu, APAP‐Sul), and NAPQI metabolites (APAP‐Mer, APAP‐Cys). The primary outcome was the proportion of NAPQI metabolites in urine relative to total excreted compounds. Secondary outcomes included 24‐hour AUCs for metabolites. Five crossover pairs were completed per formulation. Median 24‐hour urinary recovery was 92% (IQR: 89–95%). Fomepizole significantly reduced the mean percentage of urinary NAPQI metabolites for both immediate‐release (4.92% [SD: 0.97] vs. 1.72% [SD: 0.3], mean difference [fomepizole–control]: ‐3.20%, 95% CI: −4.45 to −1.95%, *P* = 0.0021) and modified release acetaminophen (5.62% [SD: 1.1] vs. 1.43% [SD: 0.3], mean difference [fomepizole–control]: −4.19%, 95% CI: −5.23 to −3.15%, *P* = 0.0004). Mean serum NAPQI metabolite AUCs were lower with fomepizole: 89.3 [SD: 11.1] vs. 32.5 [SD: 3.3] μmol/L*h (mean difference [fomepizole–control]: −56.8, 95% CI: −72.6 to −41.0, *P* =0.0006) for immediate‐release, and 96.7 [SD: 15.2] vs. 27.2 [SD:7.2] μmol/L*h (mean difference [fomepizole–control]: −69.5, 95% CI:–84.6 to −54.4, *P* = 0.0002) for modified‐release acetaminophen. Fomepizole, administered 2 hours after an acetaminophen overdose, reduced NAPQI formation by 60–70%, supporting further evaluation as an adjunct in overdoses where acetylcysteine may be insufficient.

**Study Highlights**

**WHAT IS THE CURRENT KNOWLEDGE ON THE TOPIC?**

Acetaminophen overdose causes hepatotoxicity through the formation of the toxic metabolite *N*‐acetyl‐*p*‐benzoquinone imine (NAPQI) via CYP2E1. Acetylcysteine is the standard treatment, but it may be less effective in delayed presentations or large ingestions. Fomepizole inhibits CYP2E1 and reduces NAPQI formation in cell and animal models. Human metabolite data are limited to case reports and one volunteer study in which fomepizole was co‐administered with acetaminophen at time zero. Direct human toxic metabolite data in clinically relevant delayed‐administration scenarios have been lacking.

**WHAT QUESTION DID THIS STUDY ADDRESS?**

Does administration of fomepizole 2 hours after acetaminophen overdose significantly reduce CYP2E1‐mediated NAPQI formation in humans for both immediate‐ and modified‐release acetaminophen preparations?

**WHAT DOES THIS STUDY ADD TO OUR KNOWLEDGE?**

In a randomized crossover human volunteer study, fomepizole given 2 hours after ≈80 mg/kg acetaminophen reduced urinary and serum NAPQI metabolites by 60–70% for both immediate‐ and modified‐release formulations. This provides controlled human data demonstrating a substantial reduction in toxic metabolite production when fomepizole is administered in a clinically relevant timeframe after ingestion.

**HOW MIGHT THIS CHANGE CLINICAL PHARMACOLOGY OR TRANSLATIONAL SCIENCE?**

This study demonstrates that fomepizole rapidly inhibits CYP2E1 in humans, resulting in large reductions in toxic acetaminophen metabolite formation even when administered 2 hours after ingestion. This effect is likely to be most relevant in high‐risk poisoning scenarios, particularly massive ingestions with high acetaminophen concentrations and modified‐release formulations, where absorption and oxidative metabolism may be prolonged. The increasing off‐label clinical use of fomepizole, together with these mechanistic human data, supports the need for prospective clinical trials to define the optimal timing, dosing strategies, and clinical effectiveness of fomepizole as an adjunct to acetylcysteine in acetaminophen overdose.

Acetaminophen (also known as paracetamol) is a widely used analgesic and antipyretic agent. Acetaminophen is safe in therapeutic doses but can cause acute liver injury in larger doses. Acute liver injury is the result of acetaminophen's toxic metabolite *N*‐acetyl‐*p*‐benzoquinone (NAPQI). Acetaminophen is primarily metabolized in the liver, with some extrahepatic metabolism in the kidney and intestine. At therapeutic doses, 90% of acetaminophen is metabolized into nontoxic glucuronide (APAP‐Glu) and sulfate (APAP‐Sul) conjugates that are excreted in urine.^1^ The remainder is either metabolized into NAPQI via cytochrome P450, predominately via CYP2E1, or excreted unchanged (~5%).^1^, ^2^ NAPQI binds irreversibly to glutathione, producing mercapturate (APAP‐Mer) and cysteine (APAP‐Cys) conjugates (Figure 1).^4^, ^5^ When glutathione stores are depleted, such as in overdose, NAPQI instead binds covalently to proteins (especially mitochondrial proteins), causing oxidative stress and eventually cell necrosis.^6^ Acetaminophen‐protein adducts (also known as APAP‐protein adducts) represent NAPQI covalently bound to cysteine residues on proteins that are released during cell lysis, and are predominantly from the liver.^7^ While urinary acetaminophen–protein adducts primarily reflect intrarenal NAPQI formation.^8^

**Figure 1:**
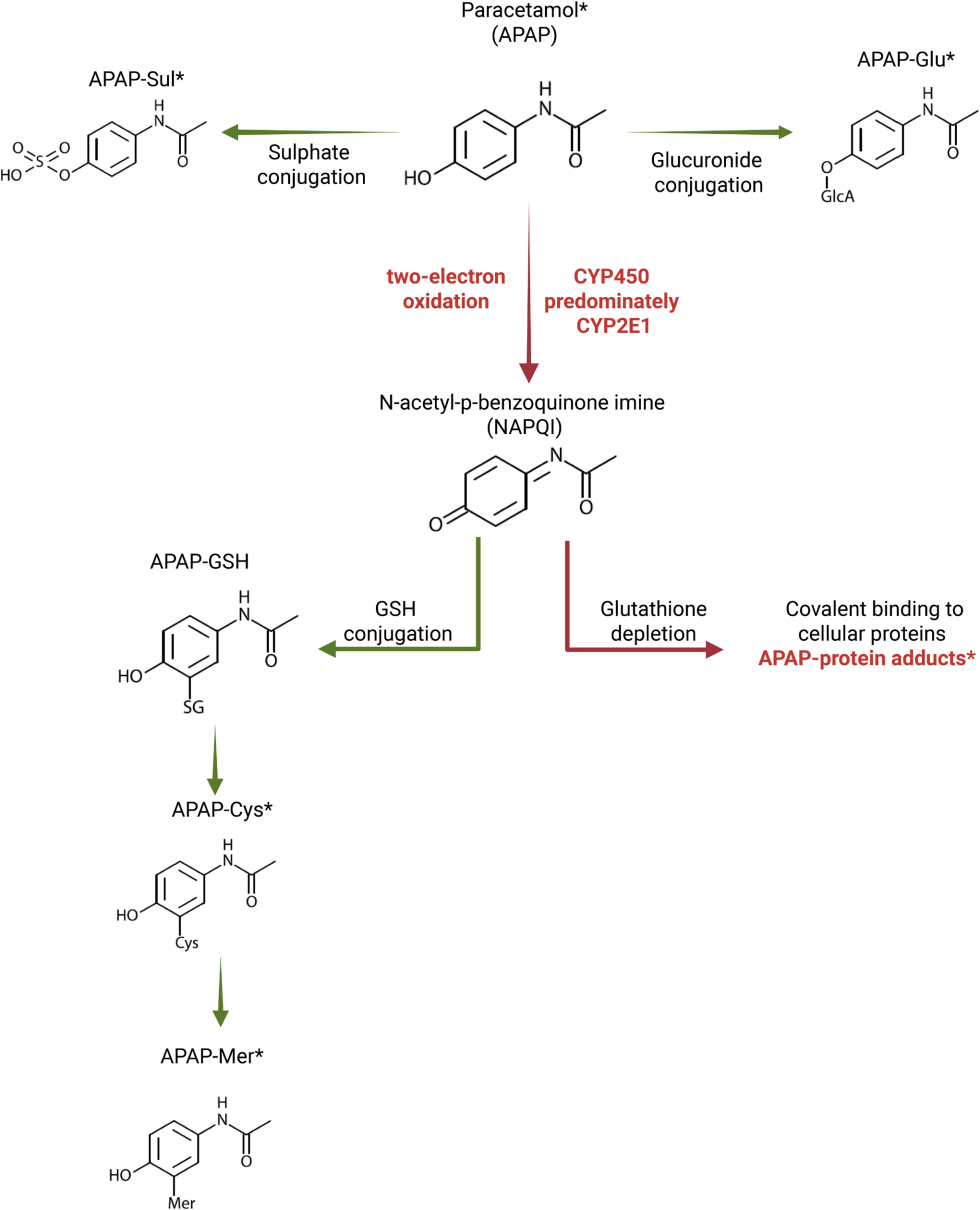
Pathways of acetaminophen (APAP) metabolism. APAP‐glucuronide (APAP‐Glu); APAP‐sulfate (APAP‐Sul); glutathione (GSH); APAP‐glutathione (APAP‐GSH); APAP‐cysteine; (APAP‐Cys); APAP‐mercapturate (APAP‐Mer). *Measured in this study. Created in BioRender. Chiew, A. (2026). Created in BioRender. Chiew, A. (2026) https://BioRender.com/3yhp1xl.^1^, ^2^, ^3^

Acetylcysteine is a highly effective antidote for acetaminophen overdose when administered within 8–10 hours of acetaminophen ingestion. In patients with immediate‐release acetaminophen overdose treated within this timeframe, reported rates of hepatotoxicity (alanine aminotransferase [ALT] or aspartate aminotransferase [AST] > 1000 U/L) are < 10%.^9^ However, patients with massive ingestions, modified‐release formulations, or delayed presentation (> 8 hours post‐ingestion) remain at increased risk of acute liver injury. Hence, there has been interest in adjunctive therapies such as fomepizole (4‐methylpyrazole). Fomepizole is traditionally used for toxic alcohol poisoning because it inhibits alcohol dehydrogenase. More recently it has been investigated as an adjunct treatment in acetaminophen toxicity, because it is also a potent inhibitor of cytochrome P450 2E1 (CYP2E1).^10^, ^11^ In addition, animal studies suggest a potential benefit via inhibition of c‐Jun N‐terminal kinase (c‐JNK) activation, thereby reducing hepatocyte necrosis in delayed presentations.^12^

Clinical data on fomepizole's ability to reduce NAPQI formation are limited. Although numerous case reports describe its use as an adjunct in acetaminophen toxicity, quantitative evidence of NAPQI inhibition remains scarce. The only human volunteer study, conducted by Kang et al., administered a supratherapeutic dose of immediate‐release acetaminophen (80 mg/kg) with intravenous fomepizole given concurrently. Fomepizole significantly reduced NAPQI production compared with controls (mean difference −3.97%, 95% CI: −2.31 to −5.63%).^13^ However, the key limitation of that study was that fomepizole was co‐administered at the time of acetaminophen ingestion, whereas in clinical practice, patients typically present 2 to 4 hours later.^14^, ^15^ Hence, this study aimed to evaluate the impact of fomepizole given 2 hours post acetaminophen ingestion in a simulated overdose model, assessing its effect in both immediate‐ and modified‐release acetaminophen formulations.

## METHODS

### Study design and setting

This was a prospective, open‐label crossover study in healthy volunteers, using a simulated subtoxic overdose model in which participants ingested ≈80 mg/kg of either immediate‐ or modified‐release acetaminophen. Participants were randomized to receive first either control (0.9% sodium chloride) or intervention (fomepizole 15 mg/kg at 2 hours post‐ingestion), with crossover to the alternate arm after a washout period. The study was conducted at the Clinical Toxicology Unit, Prince of Wales Hospital, Sydney.

### Participant selection

Healthy volunteers were recruited via advertising posters. Inclusion criteria were age > 18 years, absence of underlying liver disease, and no regular medications other than vitamins or the oral contraceptive pill. Participants with a history of chronic alcohol consumption, tobacco use, or illicit drug use were excluded. Additional exclusion criteria included pregnancy, lactation, weight > 100 kg, or body mass index (BMI) > 29, as obesity is associated with increased CYP2E1 activity.^16^ Participants received a $200 gift card upon study completion.

### Study protocol

Participants were instructed to abstain from acetaminophen for 2 weeks and from alcohol for 5 days prior to the study. Baseline liver function tests (LFTs) were obtained 3 days before study commencement, and female participants underwent a urine pregnancy test. Participants were randomized (by sequentially numbered opaque sealed envelopes) to either immediate‐release or modified‐release acetaminophen and subsequently randomized to receive control or intervention (fomepizole).

Participants received an oral acetaminophen dose of approximately 80 mg/kg, rounded to the nearest whole tablet. Immediate‐release tablets contained 500 mg acetaminophen and modified‐release tablets contained 665 mg acetaminophen (comprising 31% immediate‐release and 69% modified‐release components). On the study day, participants fasted for 6 hours prior to dosing, though water was permitted. An intravenous cannula for blood collection was inserted prior to acetaminophen dosing. Serum samples were collected at 13 time points: 0 (time of ingestion), 0.5, 1, 1.5, 2, 3, 4, 6, 8, 10, 12, 16, and 24 hours post‐ingestion. Fomepizole (15 mg/kg; Zydus Pharmaceutical®), diluted in 100 mL 0.9% sodium chloride, or control (0.9% sodium chloride), was infused over 30 minutes at 2 hours post‐ingestion. At that time, participants were permitted to eat.

Bladder emptying was done just prior to dosing. Participants then completed a 24‐hour urine collection in a 5‐L plastic container, with total volume recorded and an aliquot collected for analysis. A set of LFTs was performed 1–3 days post‐study to confirm that there was no hepatic injury. After a minimum 2‐week washout period, participants crossed over to complete the alternate arm. Volunteers were given the option to complete both immediate‐release and modified‐release arms.

This study was approved by the South Eastern Sydney Local Health District Human Research Ethics Committee (HREC: 2023/ETH00352). All participants were informed of the study design and risks and gave written consent. The study was designed and conducted in accordance with the 1975 Declaration of Helsinki.

### Analytical method for acetaminophen metabolites and APAP‐protein adducts

Serum and urine samples were collected and frozen at −80°C for batch analysis for acetaminophen, metabolites, and acetaminophen‐protein adducts (Figure 1). Acetaminophen and its metabolite concentrations were measured with an AB SCIEX Triple QuadTM 5,500 liquid chromatography with tandem mass spectrometry system (LC/MS–MS)^17^ using positive ionization mode for APAP and APAP‐d4 (internal standard), and negative ionization mode for APAP‐Glu, APAP‐Sul, APAP‐Cys, APAP‐Mer, and APAP‐Glcd3 (internal standard).^17^ Intra‐ and inter‐assay variations were assessed from the quality control samples, utilizing low and high concentrations for all metabolites. Intra‐assay variation ranged from 3.6% to 9.5%. Interassay variation ranged from 4.9% to 11.2%. The lower limit of quantitation of the assay was determined by the lowest quality control concentration measurable with a coefficient of variation < 20%. The lower limits of quantitation were 0.2 mg/L for APAP, APAP‐Glu, and APAP‐Sul, and 0.005 mg/L for APAP‐Cys and APAP‐Mer. The LC–MS/MS analyses were performed at New South Wales Pathology, Prince of Wales Hospital, Sydney.

Serum samples and urine were also analyzed for acetaminophen‐protein adducts (Figure 1) using a modification of the high‐pressure liquid chromatography‐electrochemical detection (HPLC‐EC) assay previously reported.^18^, ^19^ Assay modifications included centrifugal size‐exclusion chromatography as opposed to dialysis, and a lack of normalization to tyrosine resulted. Calibration curves were prepared over the concentration range of 0.16 to 10 nmol/mL using drug‐free serum spiked with authentic APAP‐cysteine. Standard curves were linear with regression coefficients of approximately 0.99. Samples having concentrations above the highest standard were diluted so their values fell within the range of the standard curve. Intra‐ and inter‐assay variations were assessed from the quality control samples. Intra‐assay variation ranged from 3.24 to 10.0%. Interassay variation ranged from 5.29 to 10.49%. The lower limit of quantitation of the assay was determined by the lowest quality control concentration measurable with a CV < 20%. The lower limit of quantitation for the assay was defined as 0.16 nmol/mL. The laboratory technicians were blinded to treatment allocation.

Note that APAP‐Cys refers to the glutathione‐derived metabolite, and acetaminophen‐protein adducts are a distinct pool of APAP‐Cys that is released from the protein fraction of serum or plasma following protease enzyme treatment.^20^

### Outcomes

All LC–MS/MS data were transformed from mass to molar concentrations before analysis was performed. The primary outcome was the fraction of total oxidative acetaminophen metabolites (APAP‐Cys + APAP‐ Mer)/(total metabolites + unchanged acetaminophen) in a 24‐hour urine collection following acetaminophen ingestion. This reflects the fraction of ingested acetaminophen metabolized by cytochrome P450.

The secondary outcomes included:

- Area under the serum concentration‐time curve from 0 to 24 hours of acetaminophen metabolites and acetaminophen‐protein adducts.
- Urine acetaminophen‐protein adduct concentration in a 24‐hour urine collection.

### Statistical analysis

Data are presented as mean and standard deviation for normally distributed data and median and range or interquartile range (IQR) for non‐normally distributed data. Urine concentrations of metabolites were compared between groups using the two‐tailed paired *t*‐test. Area under the time concentration curve (AUC) from 0 to 24 hours was calculated for acetaminophen and metabolites using the trapezoidal method. Area under the curve (AUC) represents the total drug and metabolite exposure and was utilized as a means of analyzing fomepizole's effectiveness in decreasing the oxidative metabolism of acetaminophen and its effect on the non‐toxic metabolite production. Urinary acetaminophen–protein adduct concentrations were right‐skewed and analyzed following logarithmic transformation, with paired comparisons performed on log‐transformed data and results presented as geometric means. All analyses were performed using GraphPad PRISM software version 10.2.1.

Sample size calculation was based on a previous healthy human volunteer study that found the percentage excretion of toxic urine metabolites following an ingestion of 80 mg/kg in healthy human volunteers is 4.48% (SD: 1.1%).^13^ A significant effect of fomepizole was determined to be a 50% reduction in oxidative metabolites in the urine. With an alpha = 0.05, beta = 0.2, and power = 0.8, five volunteers were required in each intervention group. Although the sample size in this study is too small to validly test for normality, it has been argued that a paired *t*‐test is appropriate even when sample sizes are small if the effect size is large.

## RESULTS

### Patient characteristics

Ten participants were screened, with one participant excluded due to elevated transaminases. Of the nine participants (individual demographics: **Table** **S1**), one participant performed both the modified‐release and immediate‐release acetaminophen arms. Of the nine participants, six were male, and the median age was 23 years (IQR: 21–35 years), with a median weight of 79 kg (IQR: 64–92 kg). The median ingested dose for immediate‐release acetaminophen was 81 mg/kg (IQR: 78–82 mg/kg) and for modified‐release acetaminophen was 80 mg/kg (IQR: 78–84 mg/kg).

Adverse effects reported included light‐headedness in three participants following immediate‐release acetaminophen, one participant following modified‐release acetaminophen, and two participants after fomepizole administration. Additionally, three participants reported experiencing a metallic taste following fomepizole. All participants underwent liver function testing after each trial. One participant (who received modified‐release acetaminophen and fomepizole) demonstrated a mild AST elevation to 70 U/L (reference range (RR): 10–50 U/L) at 24 hours post‐ingestion, with an ALT within reference range. The rise was subsequently attributed to muscle damage due to elevated creatine kinase (CK 1828 U/L, RR: < 200 U/L), which was consistent with recent strenuous exercise.

### Primary outcome

**Figure 2:**
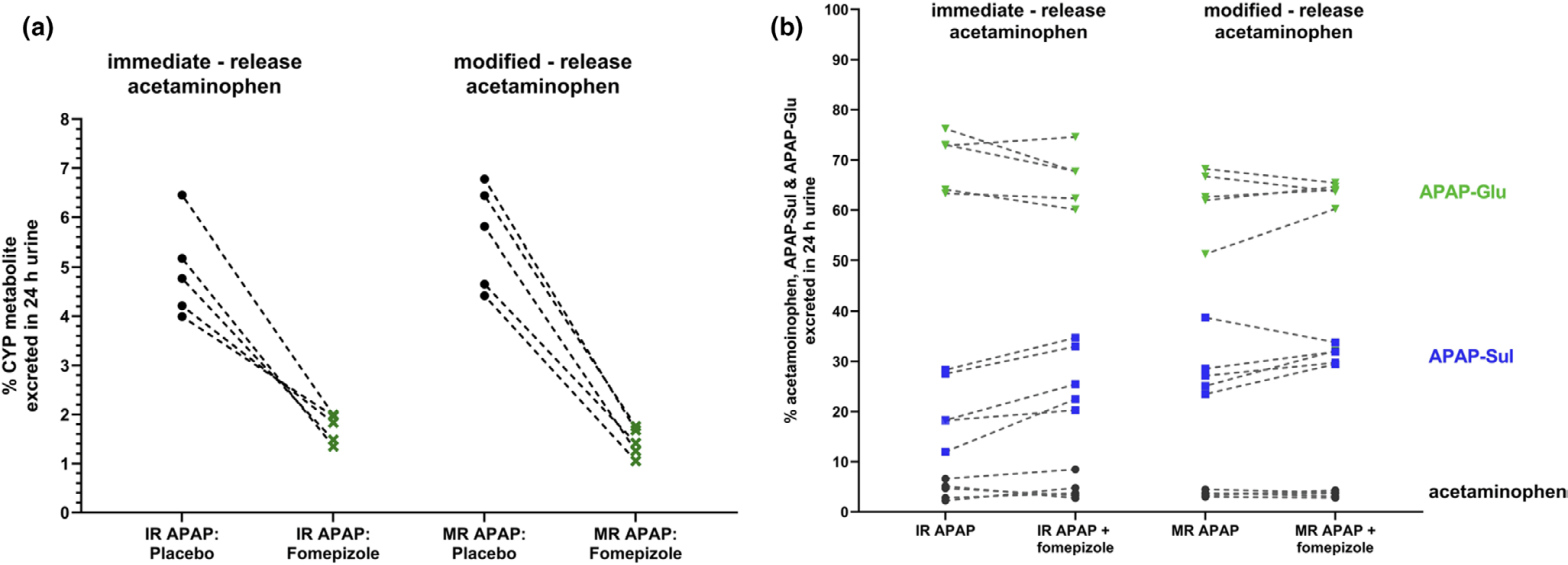
(**a**) Paired participant results for the percentage of oxidative metabolites (APAP‐Cys + APAP‐Mer) recovered in the 24‐hour urine for immediate‐release (IR) and modified‐release (MR) acetaminophen overdose models with and without fomepizole at 2 hours post‐ingestion. (**b**) Paired participant results for the percentage of acetaminophen and non‐oxidative metabolites (APAP‐Sul, APAP‐Glu) recovered in the 24‐hour urine collection for immediate‐release (IR) and modified‐release (MR) acetaminophen overdose models, with and without treatment with fomepizole at 2 h post‐ingestion of acetaminophen.

The median percentage of total administered acetaminophen recovered in the 24‐hour urine was 92% (IQR: 89–95%). The exact percentages of acetaminophen and its metabolites recovered in the 24‐hour urine are shown in **Table** **1** and **Figures** **2**. Compared to acetaminophen alone, treatment with fomepizole decreased the mean percentage of oxidative metabolites in the 24‐hour urine for both immediate‐release and modified‐release acetaminophen groups: from 4.92% (SD: 0.97) to 1.72% (SD: 0.3) (mean difference [fomepizole – control]: −3.20%, 95% CI: −4.45 to −1.95%, *P* = 0.0021) and from 5.62% (SD 1.1) to 1.43% (SD 0.3) (mean difference [fomepizole − control]: −4.19%, 95% CI: −5.23 to −3.15%, *P* = 0.0004), respectively (**Figure** **2** **a**).

**Table 1:** Mean percentage (SD) of total recovered acetaminophen and metabolites (APAP, APAP‐Glu, APAP‐Sul, APAP‐Mer, APAP‐Cys) in 24‐hour urine collections following immediate‐ and modified‐release acetaminophen overdoses, stratified by fomepizole treatment. Mean differences were calculated as fomepizole − control and are presented with 95% confidence intervals (CI) and *P* values

24‐hour urine metabolites	Immediate‐release acetaminophen			Modified‐release acetaminophen		
	Control (*n* = 5)	Fomepizole (*n* = 5)	Mean difference of % fomepizole control (95% CI, *P*)	Control (*n* = 5)	Fomepizole (*n* = 5)	Mean difference of % fomepizole vs. control (95% CI, *P*)
Mean % APAP (SD)	4.30 (1.78)	4.60 (2.30)	0.30 (−2.35 to 2.94, *P* = NS)	3.63 (0.57)	3.55 (0.60)	−0.08 (−0.94 to 0.79, *P* = NS)
Mean APAP‐Glu (SD)	69.91 (5.81)	66.50 (5.63)	−3.41 (−8.29 to 1.48, *P* = NS)	62.15 (6.65)	63.61 (2.00)	1.45 (−4.63 to 7.53, *P* = NS)
Mean APAP‐Sul (SD)	20.87 (6.94)	27.17 (6.38)	6.30 (2.54–10.07, *P* = 0.0097)	28.60 (5.99)	31.41 (1.81)	2.82 (−3.01 to 8.65, *P* = NS)
Mean APAP‐Cys (SD)	3.38 (0.53)	1.07 (0.30)	−2.31 (−3.11 to −1.52, *P* = 0.0013)	3.78 (0.45)	0.92 (0.21)	−2.86 (−3.26 to −2.46, *P* < 0.0001)
Mean APAP‐Mer (SD)	1.54 (0.57)	0.66 (0.22)	−0.89 (−1.40 to −0.38, *P* = 0.0084)	1.84 (0.73)	0.51 (0.18)	−1.33 (−2.03 to −0.62, *P* = 0.0064)

### Secondary outcomes

**Figure 3:**
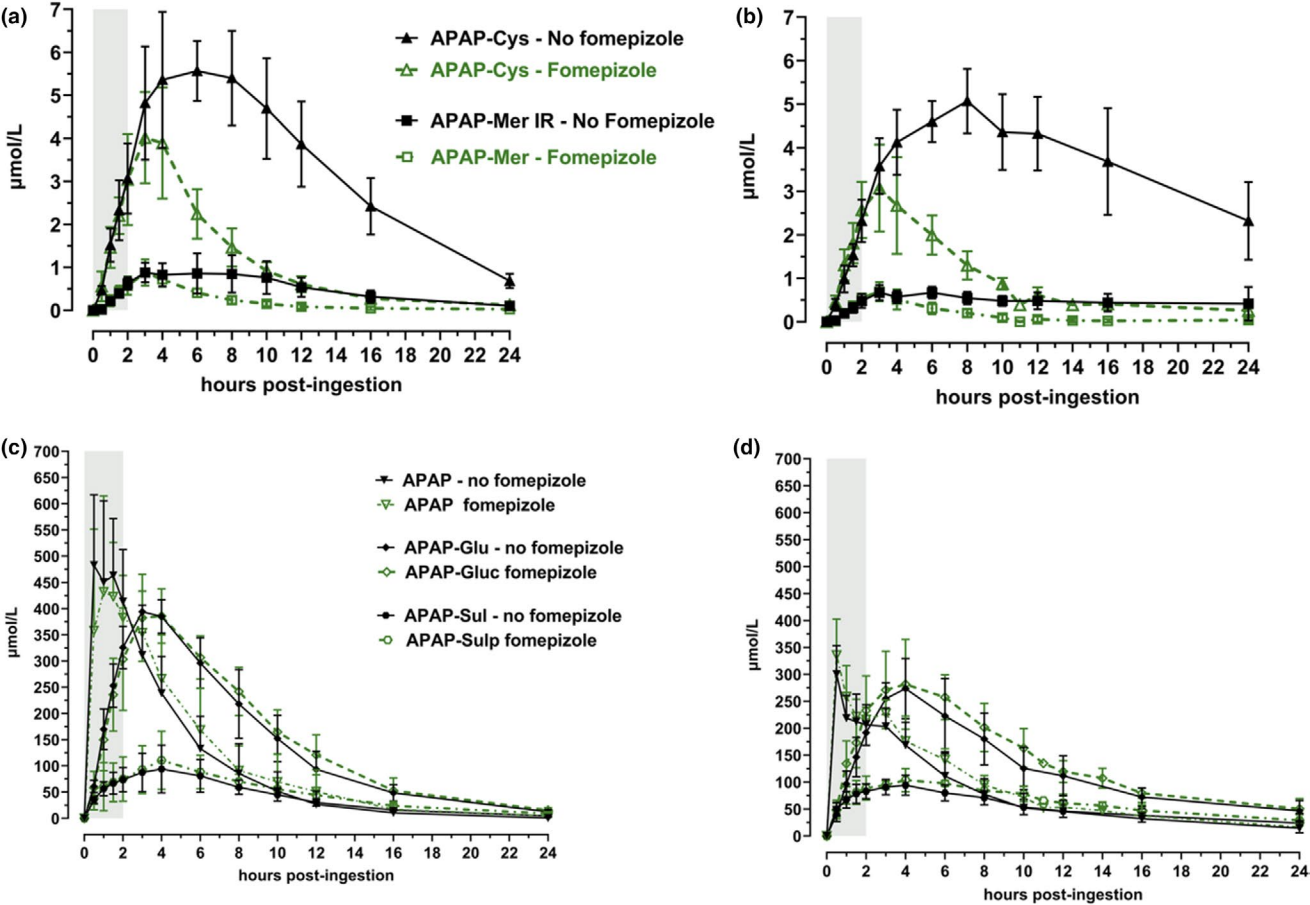
Effects of fomepizole on serum concentration‐time curves for acetaminophen and its metabolites. Concentrations shown as mean (SD), stratified by fomepizole treatment. Top panels: NAPQI metabolites (APAP‐Cys and APAP‐Mer) for immediate‐release (**a**) and modified‐release (**b**). Bottom Panels: APAP, APAP‐Glu, and APAP Sul for immediate‐release (**c**) and modified‐release (**d**). Shaded area pre‐fomepizole administration.

Areas under the serum concentration curves (μmol/L*h) (AUC) from 0 to 24 hours were calculated for acetaminophen and its metabolites (**Table** **2**, **Figure** **3**, **Figure** **S1**). The fomepizole arms had significantly lower AUCs for both NAPQI metabolites compared to control (**Table** **2**). The total mean serum NAPQI metabolites (APAP‐Cys + APAP‐Mer) AUCs were lower with fomepizole for both immediate‐release and modified release acetaminophen, 89.3 (SD: 11.1) vs. 32.5 (SD: 3.3) μmol/L*h (mean difference [fomepizole − control]: ‐56.8, 95% CI: −72.6 to −41.0, *P* = 0.0006) for immediate‐release, and 96.7 (SD: 15.2) vs. 27.2 (SD: 7.2) μmol/L*h (mean difference [fomepizole − control]: ‐69.5, 95% CI: −84.6 to −54.4, *P* = 0.0002) for modified‐release acetaminophen, control vs. fomepizole respectively. Acetaminophen, APAP‐Glu, and APAP‐Sul AUCs were unchanged (**Table** **2**, **Figure** **3**).

**Table 2:** Mean AUC (μmol/l*h) for the serum concentration curves for acetaminophen and metabolites (APAP‐Glu, APAP‐Sul, APAP‐Mer, APAP‐Cys) for both immediate‐release and modified‐release acetaminophen. Mean differences between fomepizole and control arms were calculated with 95% confidence intervals

	Immediate‐release acetaminophen			Modified‐release acetaminophen		
	Control (*n* = 5)	Fomepizole (*n* = 5)	Mean difference fomepizole ‐ control (95% CI, *P*)	Control (*n* = 5)	Fomepizole (*n* = 5)	Mean difference fomepizole ‐ control (95% CI, *P*)
Mean AUC APAP (μmol/L*h) (SD)	2362 (830.4)	2576 (915.7)	214.4, (−17.6 to 446.4, *P* = NS)	1867 (407.8)	2119 (306.2)	252, (−436.5 to 940.5, *P* = NS)
Mean AUC APAP‐Glu (μmol/L*h) (SD)	3410 (496.7)	3583 (395.5)	173.8 (−241.9 to 589.5, *P* = NS)	3024 (580.4)	3337 (566.7)	312.6, (−548 to 1173, *P* = NS)
Mean AUC APAP‐Sul	936.4 (234.7)	1143 (263.9)	207.0, (−11.6 to 425.5, *P* = NS)	1264 (234.4)	1517 (179.2)	253.6 (−36.9 to 544.1, *P* = NS)
Mean AUC (μmol/L*h) (SD) APAP‐Cys (μmol/L*h) (SD)	77.5 (7.6)	27.5 (3.9)	−50.0, (−61.4 to −38.6, *P* = 0.0003)	85.3 (13.6)	23.5 (6.4)	−61.8 (−74.5 to −49.0, *P* = 0.0002)
Mean AUC APAP‐ Mer (μmol/L*h) (SD)	11.8 (4.8)	5.0 (1.3)	−6.8 (−11.6 to −2.0, *P* = 0.0168)	11.4 (3.5)	3.7 (1.6)	−7.7 (−11.1 to −4.3, *P* = 0.0032)

**Figure 4:**
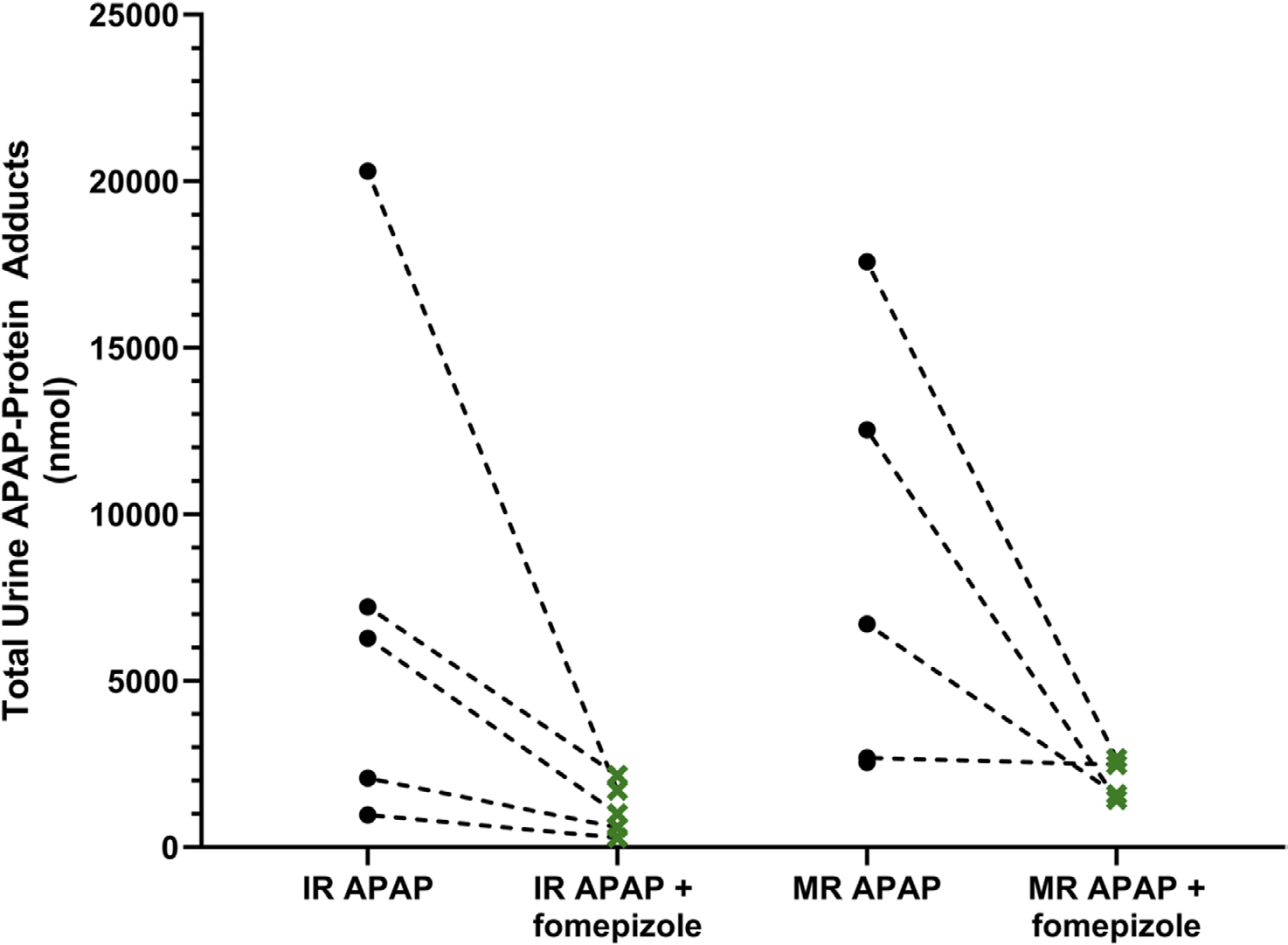
Total urine acetaminophen‐protein adduct excretion in 24‐hour urine immediate release (IR) and modified‐release (MR) acetaminophen arms, control vs. fomepizole.

Total acetaminophen–protein adducts were quantified in 24‐hour urine samples (**Figure** **4**). One MR urine result was excluded because the chromatogram showed no interpretable peak, and a reliable value could not be obtained. For immediate‐release acetaminophen preparations, the fomepizole arm showed lower urinary acetaminophen‐protein adducts. The geometric mean (SD) acetaminophen‐protein adduct excretion for control vs. fomepizole was 4507 nmol vs. 911 nmol (mean ratio: 0.2, 95% CI: 0.1 to 0.4, *n* = 5; paired *t*‐test *P* = 0.0030). Differences were not statistically significant for modified‐release preparation, geometric mean 6321 nmol vs. 1965 nmol (mean ratio: 0.2, 95% CI: 0.1 to 1.1, *n* = 4; *P* = 0.0572) (**Figure** **4**).

**Figure 5:**
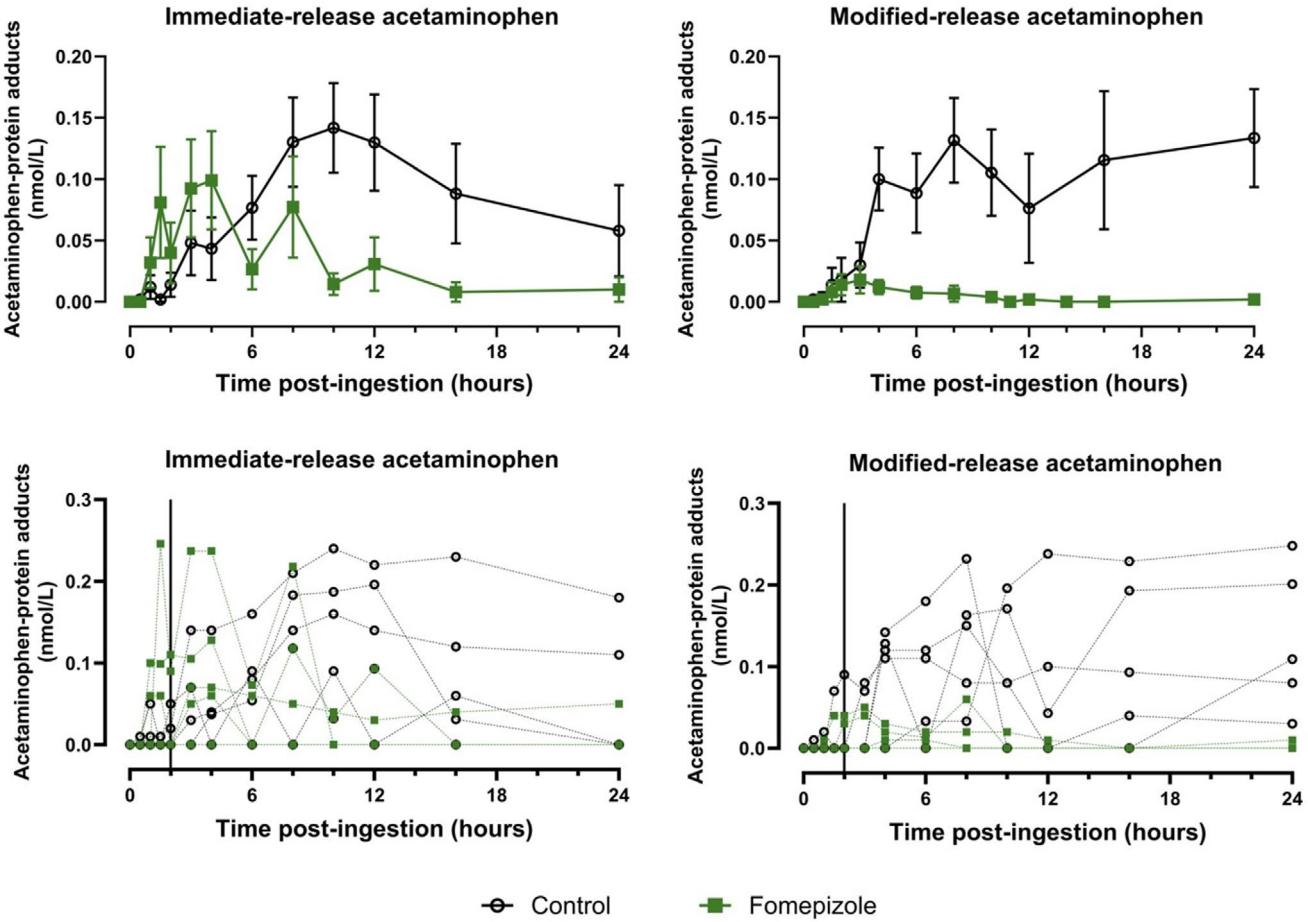
Mean (top) and Individual (bottom) serum concentration‐time curves for acetaminophen‐protein adducts. Immediate‐release arms are shown on the left and modified‐release arms on the right. The lower limit of quantitation is 0.16 nmol/L, and concentrations below this line are imprecise. Vertical lines in the bottom panels indicate the time fomepizole is given.

Acetaminophen‐protein adducts were also measured in serum samples. For most samples, adduct concentrations were less than the lower limit of quantification (0.16 nmol/L) and thus further statistical analysis was not done. However, serum samples with adduct concentrations above 0.16 nmol/mL were observed in 5/10 control arms and only one fomepizole arm. In addition, the raw pharmacokinetic data for acetaminophen‐protein adducts suggest lower NAPQI formation in the fomepizole‐treated groups (**Figure** **5**).

## DISCUSSION

In this human‐simulated acetaminophen overdose model, fomepizole administered 2 hours post‐ingestion greatly reduced the formation of oxidative (NAPQI) metabolites. There was much lower urinary excretion of NAPQI metabolites, and serum AUC further supported much lower systemic exposure. These findings provide support for a role of fomepizole in early presentations of acetaminophen overdose. This is most likely to provide clinical benefits in high‐risk scenarios where current acetylcysteine treatments are not always effective, such as massive ingestions (> 500 mg/kg) or modified‐release acetaminophen overdoses.

The use of fomepizole as an adjunct therapy in acetaminophen poisoning has increased substantially in recent years. A 10‐year retrospective observational study from the United States involving more than 1000 hospitals reported a ten‐fold rise in fomepizole use from 0.44% of acetylcysteine‐treated patients in 2013 to 6.27% (*n* = 502) in 2024, with the most pronounced increase after 2019.^11^ Despite this growing use, clinical and metabolite evidence in humans remains limited to a human volunteer study and two case reports.^13^, ^21^ Human volunteer data from Kang et al.^13^ reported an almost 90% reduction in oxidative metabolites when fomepizole was administered concurrently with immediate‐release acetaminophen in a simulated overdose model. This study, in contrast, aimed to reflect a more real‐world clinical scenario, where patients typically present hours after ingestion. This study demonstrated substantial inhibition despite delayed dosing and beyond peak acetaminophen concentrations in the immediate‐release arm. Possible explanations include delayed absorption of some of the dose, delayed Phase I (CYP) metabolism relative to Phase II conjugation. The latter may reflect early conjugation within the gut wall and/or hepatic zonation, as CYP2E1 is concentrated in pericentral (zone 3) hepatocytes, whereas conjugative enzymes are primarily periportal.^22^, ^23^ Spatial and temporal separation and delayed oxidative metabolism increase the window for fomepizole administered post‐ingestion to meaningfully inhibit CYP‐mediated NAPQI formation.

Evidence regarding the effect of fomepizole on CYP‐derived metabolite formation in acetaminophen overdose patients is limited. Recent case reports of two massive modified‐release acetaminophen overdoses (> 100 g), in which fomepizole was administered at 6.5 and 22 hours post‐ingestion, similarly demonstrated low proportions of urinary CYP‐derived metabolites (~5%).^21^ In therapeutic dosing, CYP‐mediated metabolites account for ~5–10% of urinary recovery.^1^, ^24^ In overdose, this pattern shifts; patients without liver injury maintain near‐therapeutic proportions, whereas those who develop hepatotoxicity demonstrate a higher fraction of CYP metabolites (~15%).^1^, ^24^ The low CYP‐metabolite proportions observed in our delayed‐dosing volunteer model, together with these clinical cases, indicate that fomepizole can suppress oxidative metabolism even when administered post‐ingestion. Hence, providing human metabolite evidence that CYP2E1 inhibition with fomepizole may reduce NAPQI formation in high‐risk acetaminophen poisoning scenarios.

Analysis of non‐toxic metabolites in this study demonstrated a redistribution of acetaminophen metabolism away from oxidative pathways, with preserved glucuronidation and a modest increase in sulfation following fomepizole administration (**Figure** **2**, **Table** **1**). Sulfation, mediated predominantly by SULT1A1 and SULT1A3/4,^25^ is a high‐affinity, low‐capacity pathway reliant on inorganic sulfate derived from the oxidation of sulfur‐containing amino acids and dietary sulfate.^26^ Sulfate is consumed following acetaminophen ingestion, thereby limiting sulfation's capacity. Furthermore, insufficient sulfate availability has been proposed to redirect acetaminophen biotransformation toward alternative pathways, including CYP‐mediated oxidation.^27^ As sulfate is also the terminal oxidation product of cysteine, the obligate precursor of glutathione, sulfation and glutathione synthesis are metabolically coupled and competitive.^27^, ^28^ Clinical data from two acetaminophen overdose cohorts support the clinical relevance of this pathway. These studies reported that both lower proportions of APAP‐Sul and higher APAP‐Cys/APAP‐Sul ratios were strongly associated with the subsequent development of acute liver injury.^29^, ^30^ Hence, the increased sulfation observed after fomepizole in the current study in the immediate‐release acetaminophen arm shows that, by potentially preserving cysteine and glutathione availability, this allows for increased metabolism via sulfation.

Fomepizole was associated with a reduction in oxidative acetaminophen metabolite formation following both immediate‐release and modified‐release acetaminophen ingestion. The magnitude of inhibition was greater in the modified‐release group, with a mean reduction of 4.19% compared with 3.20% in the immediate‐release group. This may be explained as the modified‐release formulations contain a substantial sustained‐release component (approximately 69%), resulting in prolonged gastrointestinal absorption and extended substrate availability for oxidative metabolism. In contrast, immediate‐release acetaminophen is typically absorbed within approximately 1 hour.^31^, ^32^ Hence, the inhibitory effect of fomepizole is likely to be more pronounced in clinical scenarios characterized by ongoing acetaminophen absorption, including modified‐release formulations, large immediate‐release ingestions, and conditions associated with delayed gastric emptying, such as co‐ingestion of drugs that impair gastrointestinal motility.

Acetaminophen‐protein adducts represent NAPQI covalently bound to protein cysteine residues, released into the blood.^18^ These adducts are highly specific markers of acetaminophen‐induced liver injury; however, low concentrations still form after therapeutic or sub‐toxic acetaminophen doses, even though glutathione is not yet depleted.^33^, ^34^, ^35^ In this study, acetaminophen–protein adduct concentrations remained below the lower limit of quantification in most volunteers. However, an observed trend toward lower concentrations following fomepizole administration again suggests a reduction in NAPQI‐adduct formation (**Figure** **5**).

Urinary acetaminophen–protein adduct concentrations varied between individuals in this study (**Figure** **4**). This variability may reflect differences in the origin and interpretation of urinary adducts compared to serum acetaminophen protein adduct concentrations. In a study by Curry et al. of acetaminophen poisoned patients comparing serum and urine adduct concentrations, 68% of 557 urine samples from 168 individuals contained no detectable adducts, even when corresponding serum concentrations were present and sometimes markedly elevated.^8^ As CYP enzymes have been found to be expressed in proximal renal tubular cells of humans.^8^, ^36^ The authors propose that urinary acetaminophen‐protein adducts arise predominantly from renal generation of NAPQI and local adduct formation within the kidney, rather than from the urinary elimination of hepatic acetaminophen–protein adducts. Thus, detection of urinary adducts likely reflects local renal oxidative metabolism and tubular protein shedding, rather than systemic clearance of serum‐derived adducts.

In our study, the difference in urine adducts was statistically significant in the immediate‐release arm but not the modified‐release arm, although the later had one less participant due to collection issues. There was a consistent trend toward lower urinary acetaminophen–protein adduct concentrations following fomepizole administration. This pattern may indicate that fomepizole reduces renal NAPQI formation as well as hepatic NAPQI formation, supporting a potential role for cytochrome P450 2E1 inhibition in limiting renal oxidative metabolism.

## LIMITATIONS

There are several important limitations to this study. As a human volunteer trial, only sub‐toxic doses of acetaminophen were administered, which limits the ability to extrapolate these findings to patients with clinically significant or massive acetaminophen overdoses. In addition, fomepizole was evaluated only when administered 2 hours after acetaminophen ingestion. In clinical practice, patients present across a wide range of time intervals, and the effectiveness of fomepizole may vary substantially depending on the degree of ongoing acetaminophen absorption and the extent of cytochrome P450 2E1 induction at the time of treatment. Further research is therefore required to determine the effect of further delays on the efficacy and pharmacokinetic effects of fomepizole.

As subtoxic doses of acetaminophen were used, another limitation of this study is that we could not examine the effect of fomepizole on oxidative stress or downstream mechanisms of acetaminophen‐induced hepatotoxicity, such as activation of the c‐Jun N‐terminal kinase pathway. Preclinical studies suggest that fomepizole may inhibit c‐Jun N‐terminal kinase activation, thereby reducing hepatocellular necrosis in delayed presentations.^12^ Studies in acetaminophen overdose patients will be needed to examine the effects on these pathways.

Furthermore, only a single dose of fomepizole was administered in this study. Patients with sustained or prolonged acetaminophen absorption, such as those who ingest modified‐release formulations, co‐ingest medications that slow gastrointestinal motility, or take massive acetaminophen overdoses, may require repeated dosing to maintain inhibition of cytochrome P450 2E1 over time. Evaluation of repeated dose fomepizole regimens, including dosing at 12 hours or later, is therefore warranted to determine whether extended enzyme inhibition confers additional protection against hepatotoxicity in real‐world overdose scenarios.

## CONCLUSION

This study provides mechanistic human evidence that fomepizole can decrease the metabolism of NAPQI via CYP 2E1 inhibition, supporting its use as an adjunct in selected high‐risk acetaminophen poisoning scenarios. The observed metabolic effects, together with emerging clinical data, suggest that fomepizole may offer particular benefit in circumstances characterized by sustained or ongoing acetaminophen absorption, including “massive” acetaminophen ingestions, particularly of modified‐release formulations. However, the use of sub‐toxic doses, restriction to early administration, and absence of clinical endpoints limit direct extrapolation to patient care. Further clinical and translational studies are required to define optimal timing, dosing strategies, and the real‐world effectiveness of fomepizole.

## FUNDING

Angela L Chiew is supported by an NHMRC Investigator Grant (2022/GNT2016380), which funded metabolite assays and participant gift cards.

Phebra Pty Ltd. covered the cost of fomepizole (Antizol, AUST R 263913) but has no ownership of the data or publications.

## CONFLICT OF INTEREST

Laura James conducted the acetaminophen‐protein adduct assay. Dr Laura James has an ownership interest in Acetaminophen Toxicity Diagnostics, LLC. Both UAMS and Dr James have a financial interest in the technology utilized in this publication. These financial interests have been reviewed and approved in accordance with the UAMS conflict of interest policies. All other authors declared no competing interests for this work.

## AUTHOR CONTRIBUTIONS

S.A., A.L.C., and N.A.B. wrote the manuscript; A.L.C. designed the research; S.A. and A.L.C. performed the research; S.A. and N.A.B. analyzed the data; P.S., L.P., L.J., and M.R.M. contributed new reagents/analytical tools. All authors met authorship criteria, contributed to the work, and approved the final version of the manuscript.

## Supporting information


**Figure S1:** cpt70379‐sup‐0001‐Figure S1.docx

**Table S1.** cpt70379‐sup‐0002‐Table S1.docx

## Data Availability

De‐identified individual participant data underlying the results reported in this Article may be made available by the corresponding author upon reasonable request.

## References

[1] Prescott, L. Kinetics and metabolism of paracetamol and phenacetin. Br. J. Clin. Pharmacol. 10(S2), 291S–298S (1980). 7002186 10.1111/j.1365-2125.1980.tb01812.x PMC1430174

[2] McGill, M.R. & Jaeschke, H. Metabolism and disposition of acetaminophen: recent advances in relation to hepatotoxicity and diagnosis. Pharm. Res. 30, 2174–2187 (2013). 23462933 10.1007/s11095-013-1007-6 PMC3709007

[3] McGill, M.R. & Hinson, J.A. The development and hepatotoxicity of acetaminophen: reviewing over a century of progress. Drug Metab. Rev. 52, 472–500 (2020). 33103516 10.1080/03602532.2020.1832112 PMC8427730

[4] Akakpo, J.Y. *et al*. 4‐Methylpyrazole protects against acetaminophen hepatotoxicity in mice and in primary human hepatocytes. Hum. Exp. Toxicol. 37, 1310–1322 (2018). 29739258 10.1177/0960327118774902 PMC6482816

[5] Link, S.L. , Rampon, G. , Osmon, S. , Scalzo, A.J. & Rumack, B.H. Fomepizole as an adjunct in acetylcysteine treated acetaminophen overdose patients: a case series. Clin. Toxicol. (Phila.) 60, 472–477 (2022). 34709101 10.1080/15563650.2021.1996591

[6] Ramachandran, A. & Jaeschke, H. Mechanisms of acetaminophen hepatotoxicity and their translation to the human pathophysiology. J Clin Transl Res 3, 157–169 (2017). 28670625 10.18053/jctres.03.2017S1.002 PMC5489132

[7] Hoffmann, K.J. , Streeter, A.J. , Axworthy, D.B. & Baillie, T.A. Identification of the major covalent adduct formed in vitro and in vivo between acetaminophen and mouse liver proteins. Mol. Pharmacol. 27, 566–573 (1985). 3990678

[8] Curry, S.C. *et al*. Prolonged acetaminophen‐protein adduct elimination during renal failure, lack of adduct removal by hemodiafiltration, and urinary adduct concentrations after acetaminophen overdose. J. Med. Toxicol. 11, 169–178 (2015). 25288219 10.1007/s13181-014-0431-2 PMC4469721

[9] Smilkstein, M.J. , Knapp, G.L. , Kulig, K.W. & Rumack, B.H. Efficacy of oral N‐acetylcysteine in the treatment of acetaminophen overdose. Analysis of the national multicenter study (1976 to 1985). N. Engl. J. Med. 319, 1557–1562 (1988). 3059186 10.1056/NEJM198812153192401

[10] Mullins, M.E. , Yeager, L.H. & Freeman, W.E. Metabolic and mitochondrial treatments for severe paracetamol poisoning: a systematic review. Clin. Toxicol. (Phila.) 58, 1284–1296 (2020). 32762579 10.1080/15563650.2020.1798979

[11] D'Aloia, M. *et al*. Trends in fomepizole use for acetaminophen poisoning in the United States; 2013–2024. J. Med. Toxicol. 21, 404–408 (2025). 40745148 10.1007/s13181-025-01091-8 PMC12511483

[12] Akakpo, J.Y. *et al*. Delayed treatment with 4‐methylpyrazole protects against acetaminophen hepatotoxicity in mice by inhibition of c‐Jun N‐terminal kinase. Toxicol. Sci. 170, 57–68 (2019). 30903181 10.1093/toxsci/kfz077 PMC6592188

[13] Kang, A.M. *et al*. The effect of 4‐Methylpyrazole on oxidative metabolism of acetaminophen in human volunteers. J. Med. Toxicol. 16, 169–176 (2020). 31768936 10.1007/s13181-019-00740-z PMC7099124

[14] Chiew, A.L. , Isbister, G.K. , Page, C.B. , Kirby, K.A. , Chan, B.S.H. & Buckley, N.A. Modified release paracetamol overdose: a prospective observational study (ATOM‐3). Clin. Toxicol. (Phila.) 56, 810–819 (2018). 29451045 10.1080/15563650.2018.1439950

[15] Chiew, A.L. , Isbister, G.K. , Kirby, K.A. , Page, C.B. , Chan, B.S.H. & Buckley, N.A. Massive paracetamol overdose: an observational study of the effect of activated charcoal and increased acetylcysteine dose (ATOM‐2). Clin. Toxicol. (Phila.) 55, 1055–1065 (2017). 28644687 10.1080/15563650.2017.1334915

[16] van Rongen, A. *et al*. Morbidly obese patients exhibit increased CYP2E1‐mediated oxidation of acetaminophen. Clin. Pharmacokinet. 55, 833–847 (2016). 26818482 10.1007/s40262-015-0357-0 PMC4916199

[17] An, J.H. , Lee, H.J. & Jung, B.H. Quantitative analysis of acetaminophen and its six metabolites in rat plasma using liquid chromatography/tandem mass spectrometry. Biomed. Chromatogr. 26, 1596–1604 (2012). 22674624 10.1002/bmc.2737

[18] Muldrew, K.L. *et al*. Determination of acetaminophen‐protein adducts in mouse liver and serum and human serum after hepatotoxic doses of acetaminophen using high‐performance liquid chromatography with electrochemical detection. Drug Metab. Dispos. 30, 446–451 (2002). 11901099 10.1124/dmd.30.4.446

[19] James, L.P. *et al*. Pharmacokinetics of acetaminophen‐protein adducts in adults with acetaminophen overdose and acute liver failure. Drug Metab. Dispos. 37, 1779–1784 (2009). 19439490 10.1124/dmd.108.026195 PMC2712440

[20] Davern, T.J. 2nd *et al*. Measurement of serum acetaminophen‐protein adducts in patients with acute liver failure. Gastroenterology 130, 687–694 (2006). 16530510 10.1053/j.gastro.2006.01.033

[21] Chiew, A.L. , McArdle, K. , Stathakis, P. & Isbister, G.K. “Massive” modified‐release paracetamol overdose: fomepizole use and measured CYP metabolites. Toxicol Commun 9, 2594956 (2025).

[22] Kietzmann, T. Metabolic zonation of the liver: the oxygen gradient revisited. Redox Biol. 11, 622–630 (2017). 28126520 10.1016/j.redox.2017.01.012 PMC5257182

[23] Hinson, J.A. , Roberts, D.W. & James, L.P. Mechanisms of acetaminophen‐induced liver necrosis. Handb. Exp. Pharmacol. 196, 369–405 (2010). 10.1007/978-3-642-00663-0_12 PMC2836803 20020268

[24] Prescott, L.F. & Wright, N. The effects of hepatic and renal damage on paracetamol metabolism and excretion following overdosage. *A pharmacokinetic study* . Br. J. Pharmacol. 49, 602–613 (1973). 4788034 10.1111/j.1476-5381.1973.tb08536.x PMC1776613

[25] Yamamoto, A. *et al*. Sulphation of acetaminophen by the human cytosolic sulfotransferases: a systematic analysis. J. Biochem. 158, 497–504 (2015). 26067475 10.1093/jb/mvv062 PMC4819960

[26] Stipanuk, M.H. Sulfur amino acid metabolism: pathways for production and removal of homocysteine and cysteine. Annu. Rev. Nutr. 24, 539–577 (2004). 15189131 10.1146/annurev.nutr.24.012003.132418

[27] Li, J. , Chiew, A.L. , Isbister, G.K. & Duffull, S.B. Sulfate conjugation may be the key to hepatotoxicity in paracetamol overdose. Br. J. Clin. Pharmacol. 87, 2392–2396 (2021). 33179287 10.1111/bcp.14642

[28] Galinsky, R.E. & Levy, G. Evaluation of activated charcoal‐sodium sulfate combination for inhibition of acetaminophen absorption and repletion of inorganic sulfate. J. Toxicol. Clin. Toxicol. 22, 21–30 (1984). 6492228 10.3109/00099308409035079

[29] Vliegenthart, A. *et al*. Circulating acetaminophen metabolites are toxicokinetic biomarkers of acute liver injury. Clin. Pharmacol. Ther. 101, 531–540 (2017). 27770431 10.1002/cpt.541 PMC6099202

[30] Chiew, A.L. *et al*. Acetaminophen metabolites on presentation following an acute acetaminophen overdose (ATOM‐7). Clin. Pharmacol. Ther. 113, 1304–1314 (2023). 36919638 10.1002/cpt.2888 PMC10952325

[31] Spyker, D.A. , Dart, R.C. , Yip, L. , Reynolds, K. , Brittain, S. & Yarema, M. Population pharmacokinetic analysis of acetaminophen overdose with immediate release, extended release and modified release formulations. Clin. Toxicol. (Phila.) 60, 1113–1121 (2022). 36106921 10.1080/15563650.2022.2114361

[32] Chiew, A. , Day, P. , Salonikas, C. , Naidoo, D. , Graudins, A. & Thomas, R. The comparative pharmacokinetics of modified‐release and immediate‐release paracetamol in a simulated overdose model. Emerg. Med. Australas. 22, 548–555 (2010). 21143403 10.1111/j.1742-6723.2010.01354.x

[33] Heard, K.J. *et al*. Acetaminophen‐cysteine adducts during therapeutic dosing and following overdose. BMC Gastroenterol. 11, 20 (2011). 21401949 10.1186/1471-230X-11-20 PMC3066114

[34] Heard, K. , Green, J.L. , Anderson, V. , Bucher‐Bartelson, B. & Dart, R.C. Paracetamol (acetaminophen) protein adduct concentrations during therapeutic dosing. Br. J. Clin. Pharmacol. 81, 562–568 (2016). 26584404 10.1111/bcp.12831 PMC4767203

[35] James, L.P. *et al*. Acetaminophen protein adduct formation following low‐dose acetaminophen exposure: comparison of immediate‐release vs extended‐release formulations. Eur. J. Clin. Pharmacol. 69, 851–857 (2013). 23052410 10.1007/s00228-012-1410-7 PMC3624058

[36] Cummings, B.S. , Lasker, J.M. & Lash, L.H. Expression of glutathione‐dependent enzymes and cytochrome P450s in freshly isolated and primary cultures of proximal tubular cells from human kidney. J. Pharmacol. Exp. Ther. 293, 677–685 (2000). 10773044

