# Supplementary material for: Effects of Fomepizole on Acetaminophen Oxidative Metabolism: A Randomized, Crossover Study in Human Volunteers
Source: Clin Pharmacol Ther. 2026 Jul 14;120(3):795–805. doi: 10.1002/cpt.70379 (PMC13366655; doi:10.1002/cpt.70379)
Supplement: Supplementary file 1 — Figure S1: cpt70379‐sup‐0001‐Figure S1.docx [file CPT-120-795-s002.docx]

 **Supplementary Figure 1:** Effects of fomepizole on serum concentration-time curves for NAPQI metabolites. Concentrations shown for individuals, stratified by fomepizole treatment. Top panels: APAP-Cys Bottom Panels: APAP-Mer, Immediate-release on left and modified-release on right. Vertical line indicates timing of fomepizole.

APAP-Mer: acetaminophen mercapturate metabolite, APAP-Cys: acetaminophen cysteine metabolite
