# Supplementary material for: Effects of Fomepizole on Acetaminophen Oxidative Metabolism: A Randomized, Crossover Study in Human Volunteers
Source: Clin Pharmacol Ther. 2026 Jul 14;120(3):795–805. doi: 10.1002/cpt.70379 (PMC13366655; doi:10.1002/cpt.70379)
Supplement: Supplementary file 2 — Table S1. cpt70379‐sup‐0002‐Table S1.docx [file CPT-120-795-s001.docx]

**Supplementary Table A:** Participant characteristic and trial allocation

| Patient letter | Age | Height (cm) and weight (kg) | Acetaminophen preparation dose (g) (mg/kg) | Sequence |
| --- | --- | --- | --- | --- |
| A | 47 F | 163 cm, 63 kg | Immediate release:  5 g, 79.37 mg/kg  Modified release:  5.32 g ,84.4 mg/kg | 1: Fomepizole  2: Control  3: Control  4: Fomepizole |
| B | 20 M | 181 cm, 94 kg | Modified release:  7.315 g, 77.82 mg/kg | 1: Fomepizole  2: Control |
| C | 21 M | 183 cm, 91 kg | Modified release –  7.315 g, 80.38 mg/kg | 1: Fomepizole  2: Control |
| D | 38 F | 165 cm, 55 kg | Immediate release-  4.5 g ,81.82 mg/kg | 1: Control  2: Fomepizole |
| E | 20 M | 188 cm, 74 kg | Immediate release-  6 g, 81.1 mg/kg | 1: Control  2: Fomepizole |
| F | 22 M | 188 cm, 85 kg | Immediate release-  7 g, 82.35 mg/kg | 1: Fomepizole  2: Control |
| G | 22 M | 166 cm, 79 kg | Modified release –  6.65 g, 84.17 mg/kg | 1: Control  2: Fomepizole |
| H | 28 F | 160 cm, 64 kg | Immediate release-  5 g, 78.13mg/kg | 1: Control  2: fomepizole |
| I | 21 M | 176 cm, 93 kg | Modified release - 7.315 g, 78.66 mg/kg | 1: Control  2: Fomepizole |
